# Cervicovaginal and gastrointestinal microbiomes in gynecological cancers and their roles in therapeutic intervention

**DOI:** 10.3389/fmicb.2024.1489942

**Published:** 2024-11-27

**Authors:** Fatimah S. Alhamlan, Ismail A. Albadawi, Ahmed A. Al-Qahtani, Khalid A. Awartani, Dalia A. Obeid, Asma M. Tulbah

**Affiliations:** ^1^Department of Infection and Immunity, King Faisal Specialist Hospital and Research Centre, Riyadh, Saudi Arabia; ^2^College of Medicine, Alfaisal University, Riyadh, Saudi Arabia; ^3^Department of Pathology and Laboratory Medicine, King Faisal Specialist Hospital and Research Centre, Riyadh, Saudi Arabia; ^4^Gynecology Oncology, Department of Obstetrics and Gynecology, King Faisal Specialist Hospital and Research Centre, Riyadh, Saudi Arabia; ^5^Reproductive Medicine, Department of Obstetrics and Gynecology, King Faisal Specialist Hospital and Research Centre, Riyadh, Saudi Arabia; ^6^Organ Transplant Center of Excellence, King Faisal Specialist Hospital and Research Centre, Riyadh, Saudi Arabia

**Keywords:** cervicovaginal microbiome, gastrointestinal microbiome, sexually transmitted infection, human papillomavirus-related cancer, gynecological cancer, cervical cancer, Women’s health

## Abstract

Cancer remains a significant global health concern, and understanding factors that regulate cancer development is important. The microbiome, with its potential role in cancer development, progression, and treatment, has garnered increasing attention in recent years. The cervicovaginal and gastrointestinal microbiomes in females constitute complex biological ecosystems. Although the gut microbiome has been extensively studied, little is known about the cervicovaginal microbiome. The microbiome plays a crucial role in maintaining local microenvironments and tissue homeostasis, but dysbiosis can disrupt this fine balance and contribute to pathological ramifications leading to cancer. This review explores the current understanding of the microbiome’s correlation with gynecological cancers and highlights the potential of microbiome-based interventions to improve outcomes in these cancers. In addition, this review underscores the gaps and limitations in the literature, such as findings in specific ethnicities compared with understudied ethnicities. In addition, discrepancies in molecular techniques and terminology (microbiome vs. microbiota) used in the literature are addressed. Emerging evidence linking gynecological cancers and dysbiosis underscores microbiota as a potential target for cancer prevention and therapy. Manipulating the microbiome, such as through the use of probiotics, prebiotics, antibiotics, or vaginal and fecal transplantation, has demonstrated benefits in the treatment of chronic and inflammatory conditions. Further translational research in this field is needed to integrate the benefits of beneficial microorganisms in the fight against gynecological cancers.

## 1 Introduction

Cancer is a significant global health challenge, representing a leading cause of death and a significant barrier to increasing life expectancy (Bray et al., 2021). According to global mortality data from 2019, >75% of the 20.4 million premature deaths occurring between the ages of 30 and 70 are attributed to non-communicable diseases (WHO, 2020). Gynecological cancers, in particular, pose a significant threat to women worldwide, especially for women in low-and middle-income countries (Sankaranarayanan and Ferlay, 2006). Gynecological cancers, including ovarian, cervical, uterine, vaginal, and vulvar cancers, encompass a range of malignancies affecting the female reproductive tract (FRT) (CDC, 2024). Cervical cancer (CC) stands out as the most common gynecological cancer, accounting for a staggering 662,301 new cases and 348,874 deaths annually. CC is followed by uterine corpus cancer, with 420,368 new cases and 97,723 deaths; ovarian cancer, with 324,603 new cases and 206,956 deaths; vaginal cancer, with 18,819 new cases and 8,240 deaths; and vulvar cancer, with 47,336 new cases and 18,579 deaths (IARC/WHO, 2022).

Over time, advancements in techniques and equipment have driven progress in research. Initially rooted in environmental microbiome research and microbial ecology, microbiome research has expanded to encompass disciplines such as agriculture, food science, biotechnology, mathematics, plant pathology, and human medicine. A significant paradigm shift has occurred, recognizing that microorganisms and their hosts form inseparable functional units. This viewpoint acknowledges that pathogens represent only a small portion of microorganisms and that alterations in microbial diversity, known as dysbiosis, can have cascading effects on the immune system and facilitate the emergence of pathogens. The history of microbiome research highlights this shift in perspective, from viewing microbes as disease-causing organisms to recognizing their essential role in the interconnectedness of life. This perspective aligns with the “One Health” concept, emphasizing the health benefits of probiotics and prebiotics in enriching microbial communities within the human body (Berg et al., 2020; Zilber-Rosenberg and Rosenberg, 2008). These improvements have greatly benefited the characterization of microbiota and have revolutionized the field.

## 2 Microbiome vs. microbiota

Despite having a long-standing history, there is ongoing debate among scientists regarding the definition of microbiome and microbiota. In a recent comprehensive review by Berg et al. (2020) an intriguing suggestion was made to revive the original definition of the term microbiome proposed by Whipps et al. (1988). This definition, which has stood the test of time for over three decades, has been further expanded by the inclusion of two explanatory sentences that differentiate between the microbiome and microbiota, emphasizing the dynamic nature of these concepts (Berg et al., 2020). The microbiome is defined as a distinct microbial community that occupies a well-defined habitat characterized by specific physiochemical properties. It encompasses not only the microorganisms but also the entire scope of their activities, leading to the formation of unique ecological niches. The microbiome is a dynamic and interactive microecosystem that is prone to changes in both time and scale. Importantly, it is integrated within macroecosystems, playing a critical role in the functioning and overall health of eukaryotic hosts (Lederberg and McCray, 2001). By contrast, the microbiota refers to the collective assembly of microorganisms belonging to various kingdoms, including Prokaryota (Bacteria and Archaea) and Eukaryota (Protozoa, Fungi, and Algae). In simple terms, microbiota is a component of the microbiome, which encompasses more than just the microbiota itself.

It is important to emphasize that the term “microbiota” traditionally refers to the microbial community comprising bacteria only and does not include viruses. However, microbial ecosystems encompass more than just bacteria, incorporating viruses, bacteriophages, fungi, and other microorganisms that collectively contribute to maintaining a balanced ecosystem. It turned out that the choice of sequencing technology employed in a study determines the types of microorganisms studied and reported. For instance, studies utilizing 16S rRNA sequencing primarily focus on the microbiota, reporting bacterial composition only. By contrast, metagenomics studies employing shotgun sequencing sequence all nucleic acids in the sample, enabling the analysis of the broader microbiome, including bacteria, viruses, fungi, and other microorganisms. Therefore, the selection of sequencing technology in microbiome studies is important in determining the scope and breadth of the microorganisms studied, ranging from specific bacterial populations to more varied microbial communities encompassing bacteria, viruses, fungi, etc. (Minot et al., 2013).

## 3 Eubiosis vs. dysbiosis

Eubiosis is the healthy or normal state of the microbiome, where the microbial community exists in a state of balanced composition and function. By contrast, dysbiosis is characterized by alterations in the structure and/or activities of the microbiome. Although some questions have been raised about the precise application of these terms, their usage in the literature remains widespread and well-established. The concept of dysbiosis has a long history, dating back to the earliest analyses of the human gut “microflora” in the late 19th and early 20th centuries (Bidell et al., 2022). Dating back to 400 B.C., Hippocrates recognized the significance of the digestive tract, stating that “death is in the bowels” and poor digestion is the root of all troubles. In the late 19th and early 20th centuries, Ali Metchnikoff proposed that disease originates in the digestive tract when “good” bacteria lose control over “bad” ones. He termed this imbalance dysbiosis, referring to an ecosystem where bacteria no longer coexist harmoniously. In a healthy gut microbiota (eubiosis), beneficial species, primarily from the phyla Firmicutes and Bacteroidetes, prevail, whereas potentially pathogenic species, such as those belonging to the phylum Proteobacteria (family *Enterobacteriaceae*), are in a minority (Iebba et al., 2016). However, in dysbiosis, “bad bacteria” overwhelm the diminished influence of “good bacteria” (Zhang et al., 2015). Hooks and O’Malley (2017) investigated how microbiome researchers discuss dysbiosis and eubiosis and the origin of the words. To investigate the expanding literature on dysbiosis, Hooks and O’Malley analyzed >9,000 PubMed abstracts that included the MeSH term “microbiota,” which also encompasses “microbiome.” Intriguingly, they found that only 5% of these articles used the term “dysbiosis”—a stark contrast to the ubiquity of the broader microbiome-related literature. Although articles on microbiota span a diverse range of medical and environmental journals, the topic of dysbiosis seems to be largely confined to discussions on human health or animal models with direct relevance to human health. The primary medical conditions most closely associated with dysbiosis include inflammatory bowel disease (IBD), *Clostridium difficile* infections, and autoimmune disorders. In the specific context of the gut microbiome, dysbiosis is often characterized by an increased relative abundance of Proteobacteria, including the notorious *Escherichia coli* and *Klebsiella* spp. In healthy individuals, Proteobacteria levels are typically maintained at <10%; however, in patients with dysbiosis, these reach a relative abundance of 20–30% (Haenel, 1961; McBurney et al., 2019; Rinninella et al., 2019). It is worth noting, however, that there is no universally accepted “gold standard” approach for identifying dysbiosis. One metric that has gained attention is the Microbiome Health Index, which assesses the relative abundance of bacteria within the phyla Firmicutes, Bacteroidetes, and Proteobacteria. The Microbiome Health Index examines the balance between bacteria in the classes Clostridia (Firmicutes) and Bacteroidia (Bacteroidetes)—known for associating with healthy gut function—versus those in classes Bacilli (Firmicutes) and Gammaproteobacteria (Proteobacteria), which are more strongly linked to pathogenesis. This index has proven particularly useful for evaluating changes in the microbiome following antibiotic exposure (Bidell et al., 2022).

The human gut microbiota is composed of an astonishingly large number of bacterial cells, estimated to be in the range of 10^13^–10^14^ (ten trillion to One hundred trillion) cells. This vast microbial community plays a crucial role in supporting various aspects of human health and physiology (Scarpellini et al., 2015; Perez-Muñoz et al., 2017; Amabebe and Anumba, 2020).

Although the gut microbiome has been the primary focus of dysbiosis research, our knowledge of microbial imbalance in other bodily systems has been growing in recent years. Advances in non-culture-based techniques have provided valuable insights into the complexities of the human body, particularly regarding the urogenital and reproductive microbiomes. Vaginal microflora has emerged as a dynamic microenvironment that is significantly influenced by factors such as pregnancy status, menstrual cycle, sexual activity, age, and contraceptive use. However, research on the vaginal microbiome reveals significant disparities between wealthy Western countries and low-and middle-income countries (LMICs). For instance, research has shown that women of European ancestry typically exhibit a Lactobacillus-dominated microbiota, particularly *L. crispatus*, associated with healthier pregnancy outcomes. In contrast, women of African descent show a low prevalence of *L. crispatus*, with limited studies focusing on their microbiomes, especially in LMICs where preterm birth (PTB) rates are disproportionately high (Matus et al., 2023). While high-income countries report a 10% mortality rate for PTB babies, LMICs, particularly in Africa, experience rates as high as 90%. Additionally, African American women in the U.S. face a 50% greater risk of PTB compared to their white counterparts. This disparity raises questions about the role of the vaginal microbiome in PTB among women of other understudied ethnicity.

Several recent studies have shown that shifts in the vaginal microbiota, including reduced lactobacilli abundance and increased populations of facultative and anaerobic organisms, can result in bacterial vaginosis (BV). This dysbiotic state not only increases the risk of adverse outcomes, such as low birth weight, but also predisposes the host to a higher likelihood of contracting bacterial infections. Interestingly, the vaginal microbiome undergoes significant changes during pregnancy, with a decrease in microbial diversity and a dominance of *Lactobacillus* spp. However, an altered vaginal microbiota characterized by low lactobacilli abundance, particularly during pregnancy, can trigger excessive inflammation and even contribute to preterm labor. In addition to BV, other forms of vaginal dysbiosis, such as those associated with the high abundance of streptococci, staphylococci, or *Enterobacteriaceae*, as well as vaginal candidiasis and trichomoniasis, hold important implications for global health (Gonzalez et al., 2021). As researchers continue to expand the frontiers of microbiome research, a more comprehensive understanding of diverse dysbiotic states and their correlations with urogenital and reproductive health will be crucial for developing targeted interventions and improving patient outcomes (van de Wijgert and Jespers, 2017). Research has demonstrated that an imbalance in the vaginal microbiome or vaginal dysbiosis can have far-reaching consequences for health. Vaginal dysbiosis has been linked to an increased susceptibility to and transmission of human immunodeficiency virus and other STIs. It has been associated with a higher risk of pelvic inflammatory disease, preterm birth, and maternal and neonatal infections. Notably, the vaginal microbiome plays a critical role during embryo implantation. Given this important function, it is not surprising that BV, a common form of vaginal dysbiosis, is more prevalent in women struggling with infertility. In fact, studies have shown that the presence of BV is associated with reduced rates of successful conception (van de Wijgert and Jespers, 2017; Saraf et al., 2021).

Maintaining a healthy vaginal microbiome is crucial. Disturbing the delicate balance of the vaginal microbiome has far-reaching implications for reproductive and sexual health. Disturbances in this microbial ecosystem can increase susceptibility to various serious conditions (Srinivasan and Fredricks, 2008). Prior to the development of advanced molecular analysis techniques and sequencing, eubiotic and dysbiotic vaginal microbiomes were primarily distinguished through Gram staining of vaginal fluid samples. In women without BV, Gram staining would typically show an abundance of gram-positive, rod-shaped bacteria. Culture-based methods would then reveal that these dominant bacteria were lactobacilli, particularly *Lactobacillus crispatus* and *Lactobacillus jensenii*. Reliance on these traditional microscopy-and culture-based approaches provided an initial understanding of the composition of the normal vaginal microbiome. However, the advent of more sophisticated molecular tools has since enabled researchers to gain deeper insights into the complexities and dynamics of this microbial community (Vallor et al., 2001; Fredricks et al., 2005). Overall, the dominance of lactobacilli, including *Lactobacillus iners*, is considered essential for maintaining a healthy and balanced vaginal ecosystem, with lactobacilli playing a protective role against the overgrowth of potentially harmful microorganisms (Sobel, 2000). However, women with BV experience a depletion of many *Lactobacillus* spp., with the notable exception of *L. iners*. This shift in microbial composition is accompanied by the acquisition of various anaerobic and facultative bacterial species (Nugent et al., 1991). Examining vaginal fluid samples using Gram staining reveals a clear distinction between eubiotic and dysbiotic states. In individuals with BV, the characteristic gram-positive rods (lactobacilli) are replaced by a diverse array of gram-negative and gram-variable cocci and rods. Culture-based analyses of vaginal fluid from women with BV typically yielded a mixed population of bacteria, including *Gardnerella vaginalis* as well as *Prevotella*, *Porphyromonas*, *Mobiluncus*, and *Mycoplasma* spp. (Łaniewski et al., 2020). This profound shift in the vaginal microbiome, characterized by the loss of protective lactobacilli and proliferation of anaerobic and facultative bacteria, is the hallmark of BV, a condition that can have significant implications for women’s reproductive health.

**Figure 1:**
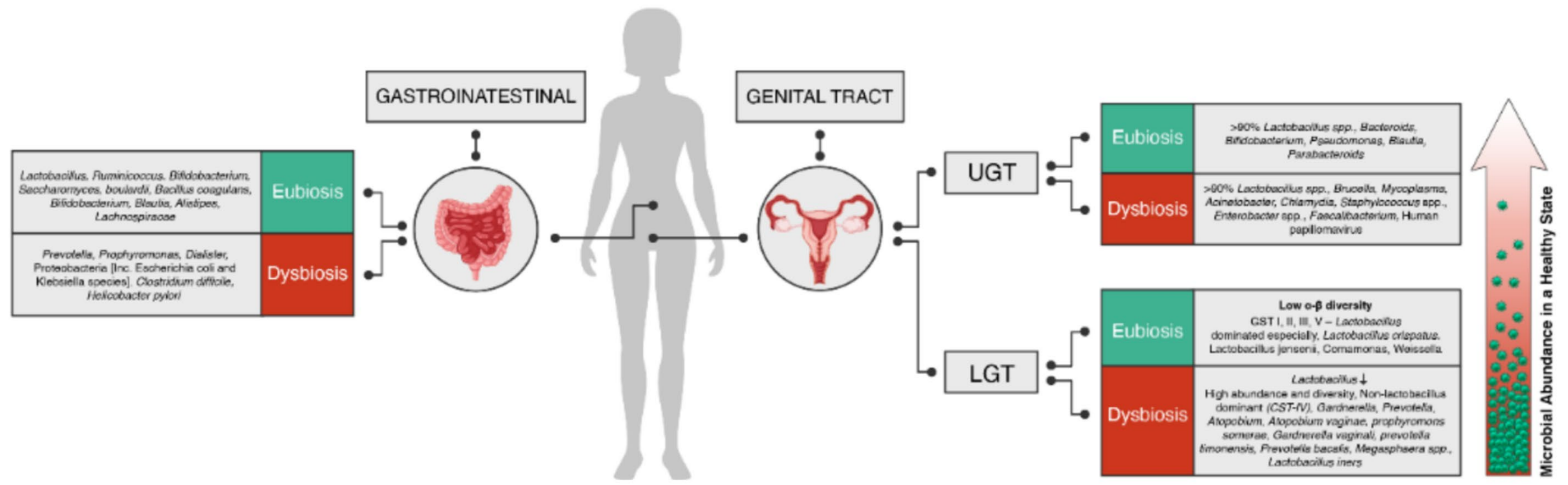
Composition of microbiota in eubiosis (green) and dysbiosis (red) in the female genital tract (upper and lower) and gastrointestinal tract. The microbiota profiles in each group are represented, highlighting the diversity and relative abundance of different microbial species or groups.

The microbiota plays a crucial role in maintaining the homeostasis of mucosal environments, including the cervicovaginal region. Health-associated *Lactobacillus* spp. are particularly important in this regard, exhibiting several beneficial functions. *Lactobacillus* spp. possess anti-inflammatory properties and help improve the barrier function of the cervicovaginal microenvironment. Lactobacilli produce lactic acid, which acidifies the local environment to a pH of <4.5. The acidic pH helps protect the host from invading pathogens while maintaining a physiological level of inflammation. Furthermore, the metabolites produced by *Lactobacillus* spp. can stimulate the host to generate antimicrobial peptides and anti-inflammatory cytokines, further strengthening mucosal defenses. By contrast, dysbiotic changes in the genital microbiome may affect various hallmarks of cancer, including chronic inflammation, barrier disruption, genomic instability, altered cell proliferation and/or apoptosis, and angiogenesis. During dysbiosis, the predominant *Lactobacillus* spp. are often replaced by a diverse mixture of anaerobic bacteria, such as *Anaerococcus*, *Atopobium*, *Dialister*, *Fusobacterium*, *Gardnerella*, *Gemella*, *Prevotella*, *Megasphaera*, *Parvimonas*, *Peptoniphilus*, *Peptostreptococcus*, *Porphyromonas*, *Shuttleworthia*, and *Sneathia*. These can induce the production of proinflammatory immune mediators and reactive oxygen species (ROS). The oxidative damage caused by ROS can have genotoxic effects on epithelial cells or alter their proliferation, potentially leading to cell apoptosis. Additionally, microbial products or metabolites may directly affect cell proliferation and disrupt the mucosal barrier. Finally, vaginal bacteria might affect angiogenesis, for example, through the stimulation of the Janus kinase–signal transducer and activator of transcription pathway and the production of angiogenic factors, such as vascular endothelial growth factor, tumor necrosis factor (TNF), antimicrobial peptides (AMPs) (Łaniewski et al., 2020). Figure 1 summarizes the known composition of the microbiota in eubiosis and dysbiosis across the female genital tract and gastrointestinal tract (Pramanick et al., 2021; Punzón-Jiménez and Labarta, 2021; Baker et al., 2018; Álvarez et al., 2021).

## 4 Cervicovaginal microbiome

Numerous studies have revealed that organs previously thought to be sterile, such as the uterus, fallopian tubes, and ovaries, can harbor microbial communities, particularly when in a disease state (Punzón-Jiménez and Labarta, 2021). The female genital tract consists of two distinct regions: the lower reproductive tract, including the vagina and cervix, and the upper reproductive tract, comprising the uterus, fallopian tubes, and ovaries. Disruptions in the vaginal flora have been associated with various gynecological diseases, including STIs, preterm birth, spontaneous abortion, pelvic inflammation, and cancer. To review the microbiome in the cervicovaginal region, it is essential to consider the gradual transition in microbial composition from the vaginal environment to the upper genital tract. The vaginal microbiome is characterized by a remarkably abundant and diverse microbial community. This contrasts with the gradually decreasing microbial presence observed as one moves from the vagina toward the cervical region and upper genital tract. As one progresses toward the cervical region and upper genital tract, the microbial composition becomes sparser, resembling a more sterile-like environment. Understanding this gradient in microbial abundance and diversity along the female genital tract is crucial for gaining a comprehensive understanding of the complex interplay between the vaginal microbiome and the health of the reproductive system (Figure 1).

### 4.1 Lower genital tract

The vaginal microbiome is important for maintaining vaginal health. Complex human microbiomes consist of colonies comprising bacteria, archaea, fungi, and viruses, each with their own genomes, metabolites, and expressed proteins. Recent evidence has revealed a connection between microbiomes and the development and progression of cancer (Xia et al., 2023). The microbiomes vary in microbial species and metabolites across different organs, and the mechanisms through which they contribute to carcinogenesis or promote cancer differ as well. Recent advancements summarized by Kroon et al. have contributed to our understanding of the microbial ecosystem in the human vagina, its impact on women’s health, and reproductive outcomes (Kroon et al., 2018). The ecology of the human vagina has been significantly enhanced by the progress in molecular and sequencing technologies. High throughput 16S rRNA gene sequencing studies have played a crucial role in examining the composition and abundance of vaginal bacterial species in women of reproductive age. Through extensive research, the vaginal microbiota has been classified into at least five major community state types (CSTs). CST I–III and CST V are predominantly occupied by *L. crispatus*, *L. gasseri*, *L. iners*, and *L. jensenii*, respectively. By contrast, CST IV consists of a more diverse, polymicrobial mixture of strict and facultative anaerobes, such as *Gardnerella*, *Atopobium*, *Mobiluncus*, and *Prevotella*, and other taxa of the order Clostridiales. Notably, CST IV is characterized by a noticeable absence of *Lactobacillus* spp. Further investigation has revealed distinct subgroups within CST IV—CST IV-A and CST IV-B. Subgroup IV-A can exhibit moderate amounts of *Lactobacillus* spp., typically *L. iners*, in addition to strict anaerobes, such as *Corynebacterium*. Conversely, CST IV-B is characterized by a higher proportion of species associated with BV (Punzón-Jiménez and Labarta, 2021). BV has consistently been linked to adverse clinical outcomes, including preterm delivery and pelvic inflammatory disease. Microscopic observation has long associated the composition of the vaginal microbiota with disease risk. The presence of *Lactobacillus* spp. is associated with protection, whereas a paucity in *Lactobacillus* spp. and the presence of a diverse set of gram-negative anaerobic species is associated with increased risk of disease. Indeed, of the 581 bacterial species detected in the vaginal microbiota, 181 were strictly anaerobic, comprising nearly one-half of Firmicutes and one-third of Bacteroidetes. These anaerobic bacteria exhibit a higher diversity at the genus level, encompassing 71 genera, with a predominance of *Prevotella*, Bacteroidetes, and numerous gram-positive anaerobic cocci species (Murphy and Frick, 2013).

A healthy vaginal microbiome is characterized by a structured microbial ecosystem, displaying lower alpha and beta diversity than other body sites. The *Lactobacillus*-dominant CSTs (I–III and V) are associated with better reproductive outcomes, whereas the more diverse CST IV, rich in anaerobic species, such as *Gardnerella*, *Prevotella*, or *Atopobium*, is linked to vaginal dysbiosis and increased disease risk (Scarpellini et al., 2015; Nugent et al., 1991; Siegel et al., 2022). A systematic review concluded that vaginal dysbiosis is a significant risk factor for early pregnancy loss in patients with Antiretroviral therapy (ART) (Skafte-Holm et al., 2021). The introduction of next-generation sequencing has facilitated the taxonomic identification of a wide range of bacterial taxa, enabling the categorization of the vaginal microbiota into various CSTs. However, further research is needed to establish diagnostic thresholds for bacterial abundance in relation to clinical outcomes in the vagina and to understand the impact of temporal changes in the vaginal microbiota (Haahr et al., 2020). Recent studies have revealed distinct microbial communities in organs other than the vagina in the FRT, such as the cervical canal, uterus, fallopian tubes, and peritoneal fluid. These findings suggest that examining the vagino-uterine microbiome could provide valuable insights into common reproductive health conditions, underscoring the need for a more comprehensive understanding of the FRT’s microbial ecosystem (Winters et al., 2019).

A recent study involving 110 women of reproductive age aimed to explore the microbial communities found in the FRT. Using 16S rRNA gene amplicon sequencing and culture techniques, the researchers identified distinct microbial communities in various locations along the reproductive tract, including the cervical canal, uterus, fallopian tubes, and peritoneal fluid. These microbial communities differed from the microbiota present in the vagina, indicating that the FRT is not a sterile environment as was previously thought. The study also identified specific microbial taxa and potential functional roles that correlated with the menstrual cycle. Certain microbial taxa were over-represented in women with adenomyosis or infertility caused by endometriosis. These findings provide valuable insights into the vagino-uterine microbiome and indicate that examining the microbial composition of the vagina or cervix can be used to detect common diseases affecting the upper reproductive tract (Chen et al., 2017).

### 4.2 Upper genital tract

The microbial composition undergoes a transition as we move toward the cervical region. The cervical microbiota exhibits a lower microbial load than the vagina. Although the vagina contains a rich array of microorganisms, the cervical canal is characterized by a sparser microbial presence. This transition is attributed to anatomical differences and the cervical mucus barrier, which restricts microbial colonization. Various studies have shown that the colonization mechanism of the upper genital tract originates from bacteria ascending from the vagina, either directly or attached to semen (Svenstrup et al., 2003). The communication between the vagina and uterus can also be influenced by factors such as peristaltic contractions, cervical fluid consistency, cervical folds, and the immune response. For instance, studies have shown that women with Bacterial Vaginosis (BV) tend to have an increased bacterial load in the upper genital tract. By contrast, some authors have argued that the vaginal and endometrial regions may have their distinct microbial communities, because certain species found in the vagina were not detected in the endometrium and vice versa. Additionally, certain gynecological procedures can transfer microbes from the vagina to the uterus (Svenstrup et al., 2003; Hansen et al., 2014). It is worth mentioning that the uterus has a relatively lower abundance of lactobacilli than the vagina and endocervix, suggesting that the abundance and composition of the microflora undergo changes along the genital tract.

The uterine cervix is a crucial anatomical structure located between the upper and lower regions of the female genital tract. It serves multiple important functions, including allowing the passage of sperm and facilitating childbirth while helping to prevent the upward movement of microorganisms into the relatively sterile uterus. Notably, the cervix is predicted to be the site for the acquisition of various STIs, such as *Chlamydia*, human papillomavirus (HPV), and human immunodeficiency virus. Healthy cervicovaginal microbiota plays a key role in maintaining the integrity of the cervical epithelial barrier and modulating the mucosal immune system. Disruptions in the composition of the cervicovaginal microbiota can lead to changes in microbial metabolites, which in turn can induce local inflammation, damage the cervical epithelial and immune barriers, and increase susceptibility to STIs and disease progression. An improved understanding of how the regulation of the cervicovaginal microbiota influences the homeostasis of the cervical microenvironment could promote advances in diagnostic and therapeutic approaches for STI-related diseases (Dong et al., 2023). For instance, the microbial community in the vagina plays a crucial role in influencing the acquisition and persistence of HPV, thereby impacting the risk of developing cervical intraepithelial neoplasia. The cervicovaginal microbiota and the host have a symbiotic relation. In a healthy female genital tract, the microbial flora is predominantly composed of *Lactobacillus* spp., which confer several important benefits to the host. Lactobacilli ferment glucose and maltose derived from vaginal epithelial cells, producing lactic acid and maintaining a vaginal pH of 3.8–4.5. The acidic microenvironment due to lactic acid helps regulate inflammation. Lactobacilli also secrete bacteriocins, which inhibit the growth of other microorganisms and allow lactobacilli to adhere to epithelial cells. This enables lactobacilli to outcompete pathogens for space and nutrients (Dong et al., 2023). While a dominant flora of lactobacilli is essential for maintaining the health of the reproductive tract. However, dysbiosis of the microbiota, characterized by a significant reduction or absence of lactobacilli and the presence of pathogenic bacteria, is associated with the development of reproductive tract-related diseases (Smith and Ravel, 2017). The microbiota of the vagina, endocervix, and uterine cavity of the same individual display a sense of continuity. The microbiota of the endocervix exhibits greater similarity to the microbiota of the vagina compared with that of the uterus and is primarily dominated by lactobacilli. However, the proportion of lactobacilli in the endocervix is lower than that in the vagina, with a notable increase in the relative abundance of *Bacteroides*, *Pseudomonas*, and *Prevotella* spp.

Positioned between the vagina and uterus, the cervix exhibits microbiota characteristics that lie between the two, making it a transformation zone for bacteria in the reproductive tract. Cervical microbiota is influenced by factors such as hormonal changes, immune responses, and interactions with the vaginal microbiota. The dominant microbial species in the cervix include *Lactobacillus* spp., such as some CSTs observed in the vagina. However, cervical microbiota have typically lower abundance and diversity than vaginal microbiota. During HPV infection, the microenvironment of the cervix undergoes changes, contributing to tumorigenesis. Depletion of vaginal lactobacilli and overgrowth of anaerobic bacteria in the cervix are associated with an increased risk of cervical cancer. The reduction in quantity or activity of lactobacilli is linked to the overgrowth of anaerobic bacteria, such as *Atopobium vaginae*, *Gardnerella*, *Fusobacterium* spp., and *Sneathia*. This dysbiosis increases the risk of carcinogenesis. After colonizing the cervix, anaerobic bacteria produce metabolites and enzymes that weaken the cervical epithelial barrier, facilitating HPV entry (Di Paola et al., 2017). Maintaining the integrity of the cervical epithelial barrier is crucial for preventing HPV from reaching the basal keratinocytes. The successful initiation of cervical cancer requires a complex interplay of factors, including microbiome dysregulation, HPV infection, and presence of inflammation. These events create an inflammatory environment that further promotes the progression of precancerous lesions and cervical cancer development (Borgdorff et al., 2016). However, emerging evidence suggests that certain bacterial species and their abundance may play a role in preventing HPV infection and aiding in viral clearance, reducing the risk of developing cancer precursor lesions (Frąszczak et al., 2022). For instance, lactic acid bacteria (LAB)-based vaccines, which have been shown to have an immunomodulatory effect, have recently attracted attention. The effectiveness of (LAB)-based vaccines increases with the shift from injections to mucosal immunization methods, such as intranasal, intravaginal, and oral routes. The magnitude of the mucosal immune response is influenced by the number of viable LAB colonies expressing E6/E7 antigens. Since the genital mucosa is a primary entry point for HPV-16, developing mucosally administered vaccines is crucial. Current research suggests that bacterial vaccines are optimal for delivering antigens to mucosal surfaces; however, using live-attenuated bacterial pathogens may pose risks for some group of patients including the immunosuppressed patients (Frąszczak et al., 2022). Continued exploration in this field is of high value, because it could lead to the development of more effective strategies to prevent and manage cervical cancer (CC) and endometrial cancer (EC). Conversely, other bacterial species may contribute to the pathological state and promote cancer complications.

Globally, CC remains the fourth most common cancer among women, despite the implementation of prevention measures, such as early screening and vaccination against HPV. Infection with high-risk HPV types, primarily HPV-16 and HPV-18, is recognized as a significant carcinogenic factor in CC development (Sung et al., 2021; Bedell et al., 2020). Although 85–90% of high-risk HPV infections can be spontaneously cleared within 6 months, a small proportion persists, leading to the development of cervical intraepithelial neoplasia, also known as squamous intraepithelial lesion, ultimately progressing to invasive CC. The persistence of high-risk HPV infection is a critical step in the multistep process of cervical carcinogenesis. Factors that contribute to the persistence of HPV infection and progression to precancerous lesions and invasive cancer are not fully understood. Emerging evidence suggests that the cervical microbiome and its dysregulation may play a crucial role in modulating the host immune response and influencing the natural history of HPV infection. Alterations in the vaginal ecosystem, characterized by a depletion of protective lactobacilli and an overgrowth of anaerobic bacteria, have been associated with an increased risk of persistent HPV infection and the development of cervical precancerous lesions (Alhamlan et al., 2021). Although global data indicate that the burden of CC is high in low-and middle-income countries, other countries are under-reported (Singh et al., 2023). Limited supply of vaccines, lack of early screening programs, accessibility to cervical screening clinics, cultural and social challenges, etc., are responsible for the high prevalence. A recent meta-analysis described recent HPV prevalence rates in the MENA region and found that the rates have continued to increase with time, especially in regions of Africa (Obeid et al., 2020).

Although cervical cancer is the most common cancer in low-and middle-income countries, EC is the most common gynecological cancer in high-income countries (Crosbie et al., 2022). The endometrium, once thought to be a sterile environment, is now known to harbor a unique microbiome. Despite the low microbial biomass, the endometrium displays a distinct microbial community, containing 100-to 10,000-fold lesser bacteria than the vaginal niche (Punzón-Jiménez and Labarta, 2021; Haahr et al., 2020). The presence of a resident endometrial microbiome has been recognized in recent years, challenging the long-held belief that the uterine cavity was a sterile milieu. This newfound understanding has opened up a new field of investigation, exploring the potential role of the endometrial microbiome in various gynecological conditions and reproductive health. The endometrial microbiome is characterized by a lower bacterial abundance than the vaginal microbiome. This disparity in microbial biomass may be attributed to the unique anatomical and physiological features of the endometrium, which presents distinct challenges for microbial colonization and survival. Despite the relatively low microbial load, the endometrial microbiome is believed to play a crucial role in maintaining endometrial health and function. Alterations in the composition and diversity of the endometrial microbiome have been associated with various gynecological disorders, including infertility, endometriosis, and EC (Walther-António et al., 2016). In fact, the bacterium *Prevotella somerae* was established as a predictive biomarker for EC (Walsh et al., 2019). Another study reported that *Acinetobacter*, *Pseudomonas*, *Cloacibacterium*, and *Escherichia* were the predominant bacteria in EC. This suggests that if there is a microbiota in the middle endometrium, it is not dominated by *Lactobacillus* as was previously concluded, and further investigation using culture and microscopy is necessary. Recently, Lu et al. found a higher abundance of the bacterial genus *Micrococcus* in the EC group than in controls with benign uterine lesions, indicating an association between dysbiosis in EC and *Micrococcus*. However, further research is needed to understand the microbial configuration of the uterine microbiota in EC. The identification of specific bacterial taxa associated with EC is an important step, but the complex interactions and mechanisms underlying the role of the microbiome in this gynecological malignancy require deeper exploration (Winters et al., 2019).

Walther-António et al. (2016). investigated the microbiome in different locations within the FRTs of patients with EC, endometrial hyperplasia (a precursor to cancer), and benign uterine conditions. Samples were collected from the vagina, cervix, fallopian tubes, ovaries, peritoneum, and urine. The microbiota present in these samples were identified using high-throughput next-generation sequencing. The results indicated that the microbiomes of the vagina, cervix, fallopian tubes, and ovaries were significantly correlated. A distinct shift in microbiome structure was observed in cases with cancer and hyperplasia compared with benign cases. Several bacterial taxa were found to be significantly enriched in samples from the EC group, including Firmicutes, Spirochaetes, Actinobacteria, Bacteroidetes, and Proteobacteria. Of particular interest, the simultaneous presence of *A. vaginae* and an uncultured representative of *Porphyromonas* spp. was associated with disease status, especially when combined with high vaginal pH. The researchers concluded that the detection of these microorganisms, in addition to high vaginal pH, was statistically associated with EC (Mahoney et al., 2022). Walsh et al. studied the influence of various patient factors, such as menopause status, body mass index, and vaginal pH, on the microbiome in the absence of EC and how these factors might contribute to the microbiome signature in EC. The results indicated that each patient factor independently affected microbiome composition. Postmenopausal status was identified as the primary driver of a polymicrobial network associated with EC, referred to as ECbiome. *Porphyromonas somerae* was identified as the most predictive microbial marker of EC. This finding was further confirmed using targeted quantitative PCR, suggesting its potential use in detecting EC in asymptomatic women at high risk (Choi and Choi, 2024).

Ovarian cancer is a highly lethal malignancy that affects women. Despite advancements in therapies, it remains the deadliest cancer of the FRT. In the United States alone, ~19,880 women were estimated to be diagnosed with ovarian cancer in 2022, with ~13,000 fatalities. Epithelial ovarian cancers make up 90% of the cases, with 60% being high-grade serous tumors that tend to spread to the peritoneal cavity. The remaining 10% consist of nonepithelial histologies, such as sex cord stromal and germ cell tumors. Malignant ovarian carcinosarcoma, a rare type of ovarian cancer with unknown origin, accounted for only 1–4% of all cases (Siegel et al., 2022; Mahoney et al., 2022). Studies have investigated the involvement of the gut and cervicovaginal microbiota in the pathogenicity of ovarian cancer. The role of the microbiome in the development of ovarian cancer and its correlation with the highly hypoxic tumor microenvironment require further exploration. Studies have shown the presence of specific bacterial species, including *Brucella*, *Mycoplasma*, and *Chlamydia*, in the ovarian microbiome of patients with cancer. Additionally, there is an increase in Proteobacteria, particularly *Acinetobacter* (Choi and Choi, 2024). *Chlamydia trachomatis*, a common STI, has been associated with an increased risk of ovarian cancer. A high presence of *Chlamydia* was found in ovarian cancer cells, and seropositivity for the chlamydial plasmid-encoded Pgp3 antibody was linked to a higher risk of ovarian cancer (Trabert et al., 2019). The correlation between chlamydial infections and ovarian cancer risk has been supported by recent meta-analyses (Hosseininasab-Nodoushan et al., 2022). Two main mechanisms have been proposed to explain how *Chlamydia* contributes to the risk of ovarian cancer. First, it induces DNA damage by producing ROS while impeding the base-excision repair pathway. Second, it evades apoptosis by inhibiting the release of mitochondrial caspase 3 and cytochrome c as well as downregulating p53 (Gulve and Rudel, 2019). Studies have indicated that *Chlamydia* can ascend to the upper female genital tract, causing inflammation and damage, potentially leading to pelvic inflammatory disease and an increased risk of ovarian cancer. Certain strains of *Lactobacillus*, such as *L. crispatus*, have shown significant bactericidal effects against *C. trachomatis*, mainly attributed to lactic acid production. This suggests that modulating *Chlamydia* through specific *Lactobacillus* spp., such as *L. crispatus*, may reduce the risk of ovarian cancer (Piao et al., 2020).

The variability in microbial communities in the fallopian tubes and ovaries across different individuals suggests that these microbiomes may be influenced by a range of factors, such as hormonal status, reproductive health, and individual-specific physiological characteristics. To investigate the changes in vaginal and endometrial microbiomes induced by variations in estrogen levels, Carosso et al. (2020) conducted a study involving controlled ovarian stimulation and progesterone supplementation. The findings revealed that in the vagina, the abundance of *Lactobacillus* decreased, whereas that of *Prevotella*, *Escherichia*, and *Shigella* increased (Carosso et al., 2020); in the endometrium, the abundance of *Lactobacillus* decreased slightly, accompanied by an increase in *Prevotella* and *Atopobium*. These results suggest that in the vagina and endometrium, microbiome composition is influenced by changes in estrogen levels. Additionally, a recent study reported that the endometrial microbiome exhibited higher transcriptional activity during the mid-secretory phase than the proliferative phase, indicating that bacterial functions are regulated in a cycle-dependent manner. Banerjee et al. focused on the association between dysbiosis of the microbiome and cancer, particularly ovarian cancer, which has high fatality rate due to asymptomatic early stages. Using their pan-pathogen array called PathoChip, in combination with capture-based next-generation sequencing, Banerjee et al. screened ovarian cancer samples as well as matched and non-matched control samples. The results revealed a unique microbiome signature consisting of viral, bacterial, fungal, and parasitic components that displayed significant relevance to ovarian cancer cases. Additionally, Banerjee et al. identified specific integration sites of viruses within the host genome of tumor samples, suggesting their potential contribution to carcinogenesis. These findings provide valuable insights for the development of targeted therapeutics aimed at combating ovarian cancers, based on the ovarian cancer microbiome signature (Banerjee et al., 2017).

The uterine microbiome may have a role in recurrent implantation failure or recurrent pregnancy loss (Benner et al., 2018). The microbiota has a significant impact on both local and systemic immunity. Recent findings, including detection of 16S rRNA in the endometrium and identification of low-biomass microbiota, have challenged the notion of the uterus as a sterile compartment. Although the concept of a “sterile womb” has traditionally focused on the *in utero* effects of microbiota on offspring and neonatal immunity, these findings suggest that the uterus is indeed a nonsterile environment. According to a recent editorial in *Lancet*, EC incidence has been rising globally. Although mortality rates have decreased overall, >40% of the countries experienced a significant increase in mortality between 1990 and 2019. Although early diagnosis improved outcome, prognosis was poor for advanced or recurrent cases (eBioMedicine, 2024). The increasing incidence of EC can be attributed to factors such as ageing population and decrease in benign hysterectomies. However, the primary cause is the rising prevalence of obesity, which presents challenges in diagnosis and treatment. Progress in understanding the molecular biology of EC has opened doors for targeted chemotherapy approaches, and ongoing clinical trials will determine their effectiveness in various disease settings (Crosbie et al., 2022). Because cervicovaginal sampling has been used for the molecular subtyping of EC to detect genetic alterations and classify tumors into four molecular subtypes, it can be used for microbiome identification. EC, the most common gynecological cancer in developed countries, is influenced by obesity, inflammation, metabolic imbalance, postmenopausal estrogen therapy, etc. The gut and vaginal microbiomes are associated with these risk factors and may contribute to EC development. Estrogen compounds can affect the vaginal microbiome, which in turn can influence endometrial hyperplasia and cancer. Specific bacteria, such as *A. vaginae* and various anaerobic bacteria, are enriched in endometrial tumors (Walsh et al., 2019). Data on uterine microbiota display discrepancies, prompting the need for further research and discussion on this contentious topic (Baker et al., 2018).

Data on the common microbiota inhabiting the uterus and fallopian tubes are limited due to challenges with assessment. The microbial communities in the fallopian tubes and ovaries exhibit significant variations across women. Unlike that in the vagina and cervix, *Lactobacillus* spp. is present at a lower proportion in the fallopian tubes and ovaries. These anatomical sites harbor a diverse range of bacteria in mildly alkaline conditions, contrasting with the acidic environment of the vagina. Studies have shown that *Lactobacillus* constitutes only a small fraction of the overall microbial composition in the fallopian tubes (Chen et al., 2017). Other bacteria such as *Bacteroides*, *Corynebacterium*, *Coproccocus*, *Hymenobacter*, *Escherichia*, or *Blaudia* have been identified in fallopian tubes, whereas *Lactobacillus*, *Corynebacterium*, *Escherichia*, or *Blaudia* have been recovered from ovary fluid, indicating considerable interindividual variability at these sites. Notably, some studies found that *Lactobacillus* spp. were not present in all fallopian tube samples, although some bacterial species were detected in all samples. The microbial signature in ovarian follicular fluid included *L. iners*, *Actinomyces* spp., *Corynebacterium aurimucosum*, *Fusobacterium* spp., *Prevotella* spp., or *Staphylococcus* spp., with colonization being more prevalent in the left ovary than the right. Understanding the composition of these microbial communities may offer insights for exploring alternative treatments for patients who would typically undergo salpingectomy for certain pathologies (Miles et al., 2017; Pelzer et al., 2018; Pelzer et al., 2011).

*Lactobacillus* spp. are the dominant bacteria in the lower reproductive tract of women, particularly those from sub-Saharan Africa. Disruptions in vaginal flora have been associated with various gynecological diseases, including STI, preterm birth, spontaneous abortion, pelvic inflammation, and cancers (Alotaibi et al., 2020; Alhamlan et al., 2016).

## 5 Gastrointestinal microbiota

Although the gastrointestinal microbiome has been extensively studied, it remains a complex and enigmatic ecosystem that continues to provide new insights. The gastrointestinal microbiome comprises approximately 100 trillion organisms. The various organisms in this ecosystem are known as the microbiome, encompassing bacteria, viruses, including phages, fungi, eukarya, and archaea. Although bacterial microbiota have been the primary focus of research due to their abundance, there are >1,000 species of bacteria in a healthy gut, mostly residing in the large intestine. The large intestine contains >400 bacterial species. The human gut microbiota contains 150-fold more genes than the human genome. Approximately 100 trillion microbes live on and inside the human body, playing key roles in various biological processes, including health and disease (Wang et al., 2017). Approximately 90% of the gut bacteria can be classified into two phyla: Bacteroidetes and Firmicutes. The remaining 10% comprises Proteobacteria, Actinobacteria, Fusobacteria, and Verrucomicrobia, in addition to a few species from the Archaea domain. In addition, the human gut microbiota contains yeasts, phages, and protists (Álvarez et al., 2021). In a state of “healthy” homeostasis, gut microbiota play crucial roles in digestion, metabolism, and immune modulation. However, a disruption in this balance, known as dysbiosis, is associated with various consequences, including infection and development of certain diseases or medical conditions (Figure 1). The dysbiosis of gut microbiota is therefore linked to various human diseases, such as anxiety, depression, hypertension, cardiovascular diseases, obesity, diabetes, IBD, and cancer. A growing body of research suggests that the microbial communities in the human body, including the gut microbiome, contribute to the development and progression of certain cancer types. Schloss hypothesized that inflammation caused by the gut microbiome can create a favorable environment for tumor formation and development. Disruptions in the microbial balance can lead to the release of ROS that can damage cells and their genetic material. Furthermore, inflammation can increase the production of growth factors and angiogenic factors, which may accelerate the spread of cancer (Ballard and Towarnicki, 2020). The composition of the gut microbiome evolves from birth and is influenced by various factors, such as diet, environment, age, and medication use. As a result, the gut microbiome of each individual is believed to be unique, akin to a fingerprint. However, there appear to be distinct characteristics that distinguish a healthy microbiome from an “unhealthy” one, often described in the context of specific patterns among commensal bacteria (Bidell et al., 2022). The gastrointestinal microbiome has been shown to play a significant role in the development and progression of certain cancer types. For example, studies have indicated that disruptions or alterations in the gut microbiota can contribute to the development of colorectal carcinoma in both genetic and carcinogenic tumorigenesis models.

Although the association between microorganisms and cancer has long been suspected, identifying specific bacterial species that can directly cause cancer has been a challenge. The International Agency for Research on Cancer has classified only *Helicobacter pylori* as a human carcinogen. In humans, *H. pylori* commonly colonizes the gastric mucosa, leading to chronic inflammation and development of gastric ulcers, which can progress to stomach cancer (Vivarelli et al., 2019; Zackular et al., 2014; Piscione et al., 2021). Additional malignancies where a single bacterial species is presumed to be the causative agent include (1) gallbladder cancer associated with chronic infection of *Salmonella enterica* serovar Typhi or Paratyphi, (2) immunoproliferative small intestine disease linked to *Campylobacter jejuni* infection, and (3) certain lymphomas associated with *Borrelia burgdorferi* or *Chlamydia psittaci* infection. In addition to bacteria, several viruses, such as Epstein–Barr virus, HPV, and hepatitis B and C viruses, cause cancer. These oncogenic bacteria and viruses can directly influence carcinogenesis through specific toxins that damage the host’s DNA or integrate oncogenes into the host’s genome, thereby disrupting normal cellular processes and promoting tumor development. The identification of these direct links between microbial pathogens and specific cancer types represents an important area of research, because it provides valuable insights into the mechanisms by which microorganisms contribute to the initiation and progression of malignancies, potentially leading to the development of targeted strategies for the early detection, prevention, and therapeutic intervention of cancer (Łaniewski et al., 2020; Garrett, 2015). For example, research on human fecal samples has identified differences in microbiome composition between patients with colon cancer and healthy individuals, including an increase in specific bacteria associated with the disease. Certain strains of bacteria were found to be present at different stages of colon cancer, contributing to cancer growth through various mechanisms, such as initiation of signaling, production of metabolites that create a tumor-friendly environment, and exertion of genotoxic effects. The microbiome’s influence on cancer development may be related to inflammation and the release of ROS and growth factors (Ballard and Towarnicki, 2020). In a meta-analysis, Zhou et al. examined the role of the gut microbiome in gynecological diseases. The study found a decrease in the richness and diversity of the gut microbiota in patients with gynecological diseases. Specific alterations were observed in patients with endometriosis and polycystic ovary syndrome. Patients with endometriosis exhibited a decrease in Shannon index, which is a measure of microbial diversity, in their gut microbiome. Patients with polycystic ovary syndrome, particularly those who were obese, showed decreased observed species, Chao1, and Shannon index, indicating a reduction in gut microbial richness and diversity. The study suggested that the gut microbiome contributes to the development of polycystic ovary syndrome through its interactions with metabolism, energy absorption, hormones, and glucose metabolism disorder. Overall, the results demonstrated that alterations in both the gut and genital microbiota are associated with major gynecological diseases. The meta-analysis revealed that the most observed results were shared alterations across diseases rather than disease-specific alterations. This suggests that the gut and genital microbiome play a common role in the pathogenesis of gynecological disorders (Zhou et al., 2024). The authors concluded that further investigation is required to identify specific biomarkers that serve as promising diagnostic tools. Studies must evaluate the mechanisms and pathways mediating gynecological diseases and the observed microbial alterations. The intestine harbors a wide array of microorganisms that contribute to a healthy environment, whereas the female genital tract typically exhibits low microbial diversity, often consisting of only one or a few types of lactobacilli. Evidence suggests the transfer of bacterial strains from the gut to the vagina. Both the gut and vagina harbor common bacterial phyla, including Firmicutes, Bacteroidetes, Proteobacteria, Actinobacteria, and Fusobacteria. The dominant gram-positive *Lactobacillus* in the healthy vaginal microbiota is believed to originate from the gut. Lactobacilli are abundant in the gut and contribute to energy, metabolic, and immunological balance. The crosstalk between bacterial strains in the gut and vagina stimulates local and systemic immune responses, impacting overall host physiology (Amabebe and Anumba, 2020). The female microbiome is interconnected through various axes within the body. The FRT microbiota interacts extensively with microbiomes in other body sites, forming complex axes of interaction. The vagina–gut axis and vagina–bladder axis as well as potential connections to the microbiome of the oral cavity highlight the correlations between the FRT and distant mucosal sites. Bacteria in the lower FRT, including *Lactobacillus* spp. and dysbiotic anaerobes, can ascend to the upper FRT. Additionally, common vaginal bacteria, such as *Lactobacillus* spp., are also found in the urinary tract microbiota, and *Lactobacillus* spp. from the vagina can even colonize the rectum. More studies have shown that the gut microbiota is increasingly recognized as a crucial endocrine organ that influences various bodily functions and distant organs throughout a woman’s life. It interacts with hormones such as estrogen, androgens, and insulin, playing a significant role in the reproductive endocrine system. An imbalance in gut microbiota composition can lead to several health issues, including gynaecological cancers. However, research on the underlying mechanisms of these microbiota-hormone interactions is still limited (Kashyap et al., 2021).

Furthermore, bacteria may spread through the bloodstream (hematogenous spread) from the oral cavity and seed the upper FRT microbiome, suggesting the presence of reservoirs of genital microorganisms outside the reproductive tract. These complex correlations between FRT and other body site microbiomes highlight the importance of considering the holistic microbiome when studying gynecological health and disease (Łaniewski et al., 2020).

### 5.1 Therapeutic approaches

Therapeutic approaches for addressing gut and vaginal dysbiosis in gynecological cancer involve a range of interventions, including fecal microbiota transplantation (FMT), vaginal microbiota transplant (VMT), probiotics, prebiotics, metabolites, hyaluronan, miRNA, and engineered bacteria and viruses (Gebrayel et al., 2022). It has been shown that the interactions between the microbiome and cancer therapies—such as surgery, radiotherapy, chemotherapy, and immunotherapy—significantly impact microbial diversity and composition. While the adverse effects of radiation and chemotherapy on gut microbiota are well-established, leading to gastrointestinal disturbances and vaginal complications, modulating the gut microbiota can mitigate these toxic effects and enhance treatment efficacy (Lin et al., 2023). In fact, strategies such as antibiotics to reduce harmful microbial populations, fecal microbiota transplantation (FMT), vaginal microbiota transplantation (VMT), and supplementation with prebiotics or probiotics may improve responses to cancer therapies. Studies have shown that the gut microbiome influences responsiveness to immunotherapy and impacts the effectiveness of cancer treatments. FMT has demonstrated promise in reducing the toxic effects associated with radiotherapy and chemotherapy, positioning it as a potential adjunct therapy for cancer treatment. However, further research and safety assessments are essential, particularly for immunocompromised patients (Baldassarre et al., 2018).

Fecal and vaginal microbiota transplantation can be used to manage female genital tract disorders associated with dysbiosis, similar to approaches used for the gut. While the mechanisms underlying these therapies are still being explored, it is believed that commensal microbes and their metabolic byproducts possess antimicrobial and immunomodulatory properties that help restore eubiosis and homeostasis. Fecal microbiota transplantation (FMT) specifically aims to modify the recipient’s gut microbiota to achieve therapeutic benefits. It involves transferring fecal material from a healthy donor to a recipient. Approved by the U.S. Food and Drug Administration for treating recurrent and refractory *Clostridium difficile* infections since 2013, FMT applications have rapidly expanded to include various extra-gastrointestinal diseases. Specific gut microorganisms identified through FMT studies have demonstrated the ability to modulate therapeutic responses. For example, certain *Bifidobacterium* spp. administered orally to mice with melanoma showed efficacy comparable to PD-L1 inhibitor treatment alone and enhanced the effectiveness of PD-L1 inhibitors when administered together. Supplementation with *A. muciniphila*, a gut commensal, restored the response to PD-1 inhibitors in mice that received FMT from patients who did not respond to PD-1 therapy (Tuniyazi and Zhang, 2023).

Vaginal microbiota transplantation (VMT) involves the transfer of whole vaginal fluid from a healthy individual with an optimal vaginal microbiota, characterized by a high abundance of Lactobacillus, to a recipient suffering from vaginal diseases. This therapeutic approach aims to restore a healthy vaginal microbiome, particularly following antibiotic treatment. For instance, VMT is used for treating bacterial vaginosis (BV), which is characterized by an overgrowth of diverse anaerobic bacteria in the vagina. Traditional treatments, including antibiotics, often lead to recurrence and only temporary changes in the vaginal microbiota. Therefore, additional treatment strategies are needed for long-term remission of BV symptoms in patients. In 2019, the first report on VMT in humans showed that four out of five women experienced remission of BV after undergoing multiple antibiotic treatments over 2 years, followed by the introduction of a small quantity of fresh vaginal fluid (Younge et al., 2019). In 2022, Yockey et al. has established an FDA-approved donor screening protocol to ensure the safety and viability of the transferred microbiota. They reported that Lactobacillus viability can be maintained for over 6 months when stored properly (Yockey et al., 2022).

Vaginal microbiome modulation also has been examined through the use of vaginal probiotic lactobacilli, such as *L. crispatus* strain CTV-05 (commonly administered as a vaginal suppository known as LACTIN-V) (Ruth and Field, 2013). Clinical trials have primarily focused on treating BV or UTIs. In a randomized placebo-controlled phase IIa clinical trial involving women with BV who received standard metronidazole treatment, 44% of the participants achieved vaginal colonization with LACTIN-V by day 28, which inhibited the growth of BV-associated bacteria, particularly *Atopobium* (*p* = 0.04). Notably, the study found that vaginal intercourse during treatment (*p* = 0.003) or precolonization of the vagina with endogenous *L. crispatus* (*p* = 0.018) reduced the likelihood of successful colonization with the probiotic *L. crispatus* strain. Another multicenter phase IIb clinical trial (NCT02766023; *n* = 228) assessing the long-term efficacy of repeated doses of LACTIN-V in preventing BV recurrence has been completed (unpublished results). Additionally, a phase II trial (*n* = 100) evaluated LACTIN-V supplementation for the prevention of recurrent UTIs and demonstrated a reduction in recurrent UTIs compared with placebo treatment (*p* < 0.01). These studies provide evidence of the potential of vaginal probiotics in modulating the vaginal microbiome and indicate their feasibility in various clinical settings.

Notably, microbiome transplantation carries a potential risk of transmitting pathogenic and opportunistic microorganisms. The safety concerns associated with this therapy are the primary limitations when considering its use in clinical settings. Therefore, thorough screening of donors is crucial to minimize the risk of exposure to infectious agents. Currently, the selection process for donors focuses on maximizing safety by excluding individuals with potential risk factors, aiming to obtain a relatively healthy vaginal microbiota characterized by a high abundance of *Lactobacillus* spp. (Tuniyazi and Zhang, 2023). It is essential to acknowledge that microbiome transplantation therapy does not provide guaranteed treatment for all disorders. Specifically, in the case of vaginal disorders, vaginal microbiome transplantation is primarily utilized for BV. However, its effectiveness in treating viral vaginosis is unknown (Lev-Sagie et al., 2019).

Probiotics represent a promising therapeutic approach for addressing gut and vaginal dysbiosis in gynecological cancer (Dunlop et al., 2023). While oral supplementation is commonly used to treat gastrointestinal dysbiosis, probiotics can also be administered intravaginally to target conditions such as bacterial vaginosis (BV) and other related disorders (Koren et al., 2012). Intravaginal administration allows for a more direct and rapid restoration of the vaginal microbiota, potentially enhancing therapeutic outcomes. Research has shown that combining antibiotics with intravaginal probiotics can improve cure rates and reduce recurrence rates of BV. For instance, studies indicate that the use of *Lactobacillus* species alongside antibiotics significantly lowers the recurrence of BV symptoms compared to antibiotics alone. They inhibit the growth of pathogenic bacteria and produce antimicrobial substances that enhance their beneficial impact. For instance, the probiotic mixture VSL#3, which comprises multiple species of *Lactobacillus*, *Bifidobacterium*, and *Streptococcus*, is utilized in the treatment of inflammatory bowel disease (IBD), irritable bowel syndrome (IBS), pouchitis, obesity, and female reproductive diseases (Rasmussen et al., 2020). These probiotic strains effectively improve gut health by modulating the microbiota and promoting a balanced microbial composition, underscoring their potential therapeutic applications in both gastrointestinal and vaginal health (Amabebe and Anumba, 2020). In addition, to address antibiotic resistance, probiotic lactic acid bacteria have emerged as alternatives to antibiotics. Lactic acid bacteria not only play a role in preserving the organoleptic profile of fermented food products but also affect the composition and diversity of intestinal microbiota. They stimulate the host’s immune system, prevent antibiotic-associated diarrhea, treat IBD and irritable bowel syndrome, alleviate lactose intolerance, lower cholesterol levels, and help prevent gastrointestinal infections, such as *C. difficile*-associated diarrhea (Amabebe and Anumba, 2020).

In addition to probiotics, prebiotics have been gaining recognition for their beneficial effects on human health. Prebiotics are non-digestible nutrients that are degraded by gut microbiota (Rowland et al., 2018). They selectively stimulate the growth and activity of specific bacteria in the colon, leading to improvement in host health. Prebiotics are classified into various types. The degradation of prebiotics by gut microbiota produces short-chain fatty acids, which have anti-inflammatory properties and can regulate several human disorders. Prebiotics are considered safe and effective, with minimal side effects, and have shown therapeutic potential in conditions such as IBD and genetically-induced obesity. However, research on prebiotics in humans is relatively limited, with most studies conducted *in vitro* or in animal models. Prebiotics, alone or in combination with probiotics, have demonstrated positive effects on the immune system–microbiota interaction and prevention of colorectal cancer (Davani-Davari et al., 2019; Ambalam et al., 2016). There are ongoing research ineptest to explore the scientific evidence regarding the impact of prebiotics, probiotics, and synbiotics on HPV infections, precancerous lesions, and various stages of cervical cancer development and treatment. In a recent review, findings suggest that higher dietary fiber intake correlates with a reduced risk of HPV infection, while specific probiotics have shown effectiveness in clearing HPV-related lesions. Additionally, prebiotics such as inulin and fructo-oligosaccharides, along with synbiotics, have been found to decrease gastrointestinal side effects in cervical cancer patients. These agents exert their effects by modulating metabolic pathways that reduce inflammation and oxidative stress, promote apoptosis, inhibit cell proliferation, and suppress oncogene activity, thereby mitigating tumorigenesis (Saha et al., 2024).

Gut microbiota-associated metabolites have emerged as crucial regulators in the development and progression of various diseases, including cancer, and are being explored as novel therapeutic strategies. These metabolites are utilized in treating local inflammation and modulating cardiometabolic and neurological disorders (Gebrayel et al., 2022). These metabolites, including short-chain fatty acids (SCFAs) like butyrate, exhibit anti-inflammatory properties and have been shown to influence various health conditions, including cancer. Their natural bioavailability, high concentrations, and tissue tolerability make them attractive candidates for clinical applications. Recent advancements in metabolomics technologies have enhanced our understanding of how these metabolites interact with cancer therapies, suggesting that direct supplementation could improve treatment outcomes. Studies indicate that gut microbiota can both reduce cancer risk and enhance the efficacy of anticancer therapies (Bultman and Jobin, 2014). Currently, research on metabolites as therapeutic agents has primarily focused on colorectal cancer, hepatocellular carcinoma, and breast cancer, with limited studies in gynecological cancers. This gap highlights the urgent need for continued investigation into the role of microbiota-derived metabolites in gynecological cancer (Descamps et al., 2019). Understanding these interactions is essential for optimizing treatment strategies and addressing dysbiosis, which can significantly impact patient outcomes.

Recent research has underscored the potential of microRNAs (miRNAs) as innovative therapeutic tools for modulating the gut microbiome, although most studies have concentrated on gastrointestinal conditions rather than gynecological cancers. miRNAs are short non-coding RNA molecules that regulate gene expression and are crucial for maintaining intestinal homeostasis. Dysregulation of miRNA interactions is linked to inflammatory bowel disease (IBD), suggesting therapeutic avenues through fecal transplantation and probiotics (Mafune et al., 2022). While preclinical studies demonstrate that gut microbiota can affect miRNA expression, the specific signatures and mechanisms involved are still being elucidated. For instance, certain probiotics can alter intestinal miRNA profiles in a species-specific manner, suggesting that targeted probiotic therapies could modulate gut health through miRNA regulation. However, the exploration of miRNAs in the context of gynecological cancers remains limited. The same applies to hyaluronan (HA), which is an essential element of the extracellular matrix (ECM) that maintains normal structural integrity and development, while also playing a key role in tissue responses during injury, repair, and regeneration. It is emerging as a promising therapeutic agent for addressing dysregulation in the microbiota-immune-gut axis; however, it has primarily been studied in gastrointestinal conditions. Recent research indicates that HA can modulate inflammatory bowel disease (IBD) by regulating immune cell recruitment and maintaining homeostasis. Its effects are mediated through receptors like CD44 and toll-like receptors (TLRs), which play roles in cellular responses during inflammation. While current studies focus on IBD and gastrointestinal disorders, exploring HA’s applications in gynecological contexts could reveal its therapeutic potential for related conditions (Gebrayel et al., 2022).

Genetically engineered microbes represent innovative therapeutic strategies for gynecological cancers, leveraging advances in synthetic biology and virotherapy to target tumor cells effectively. Genetically engineered microbes have shown significant promise as therapeutic agents, utilizing their ability to colonize tumors. Researchers are repurposing bacteria as tumor-specific delivery vehicles that can modulate the tumor microenvironment through their inherent immunogenicity and localized therapeutic payload production. For example, non-invasive bacteria like the probiotic strain *E. coli* Nissle 1917 (EcN) can be modified to secrete therapeutic proteins directly into the tumor microenvironment. One innovative approach involves engineering EcN to express a Shigella-derived type 3 secretion system, allowing for targeted delivery of therapeutic agents while minimizing systemic exposure. In preclinical models, such as colitis in mice, these engineered bacteria have demonstrated efficacy comparable to traditional systemic therapies, effectively reducing inflammation through localized action (Sakuraba et al., 2024). Oncolytic viruses also offer a promising avenue for virotherapy in gynecological malignancies by selectively infecting and replicating within cancer cells. Unlike traditional gene therapy that employs replication-incompetent viral vectors, OVs are replication-competent agents that specifically kill cancer cells while sparing healthy tissues. When OVs infect tumor cells, they release progeny virions that can spread throughout the tumor, providing a significant advantage over conventional viral vectors that lack this ability. Beyond their direct cytopathic effects—known as oncolysis—OVs can deliver therapeutic transgenes to enhance their anti-cancer properties (Vaghefi et al., 2021).

**Figure 2:**
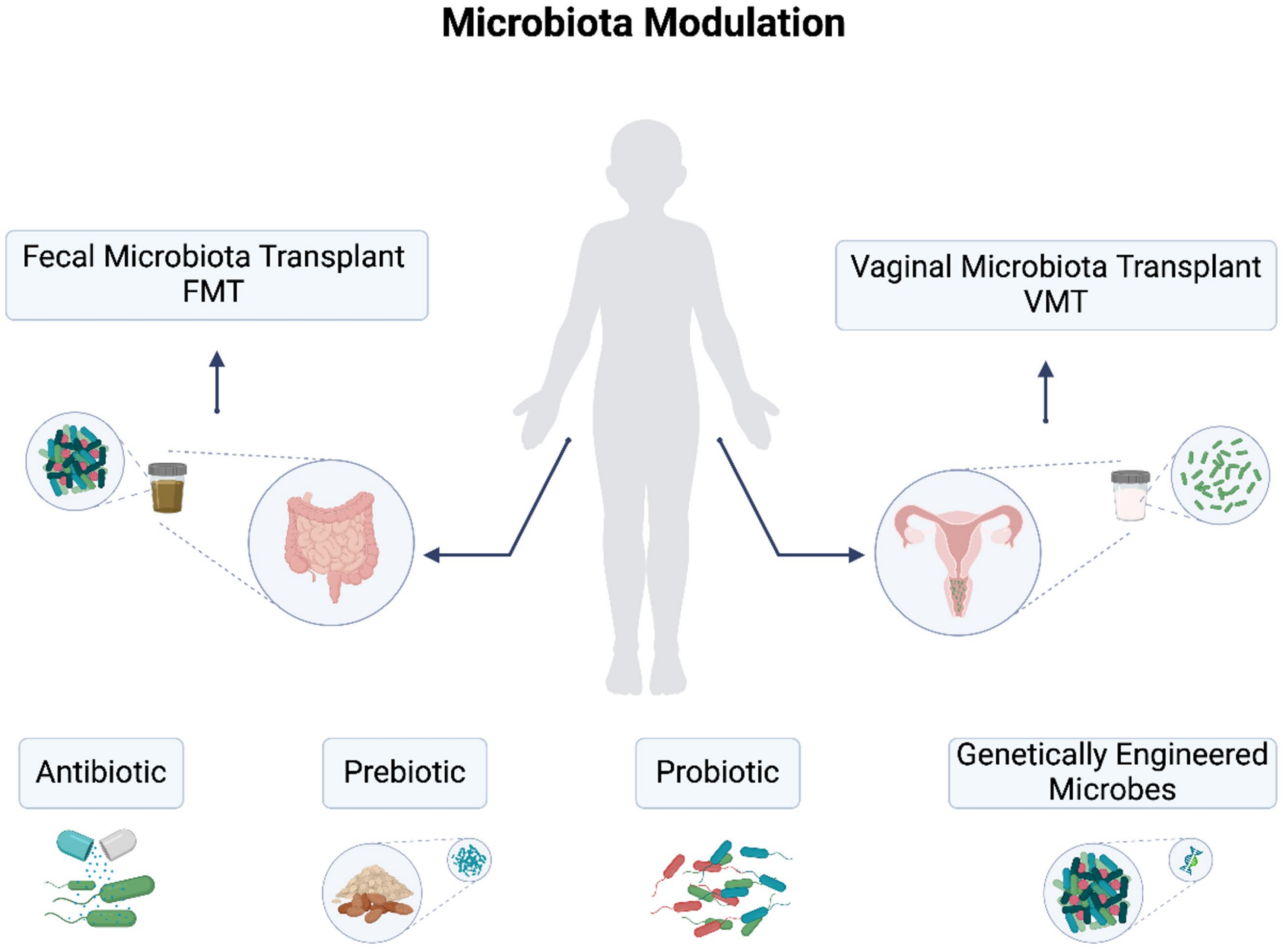
Therapeutic approaches for gut and vaginal dysbiosis in gynecological cancers. Approaches include fecal microbiota transplantation (FMT), vaginal microbiota transplant (VMT), probiotics, prebiotics, antibiotics, and engineered bacteria and viruses.

Historical clinical trials have illustrated the potential of OVs in treating gynecological cancers. For instance, a trial in the 1950s involving various adenovirus serotypes showed that over 50% of cervical cancer patients experienced marked to moderate local tumor responses, although systemic responses were limited. Subsequent studies further demonstrated efficacy; in 1965, treatment with Newcastle disease virus resulted in tumor shrinkage in a cervical cancer patient, while a 1988 trial using mumps virus led to complete clinical resolution in several patients with ascites or pleural effusion. As research continues to evolve, the integration of genetically engineered microbes may provide novel therapeutic strategies for gynecological cancers (Mazmanian et al., 2010). Figure 2 illustrates the therapeutic approaches for microbiota modulations.

## 6 Limitation

The existing literature provides limited insights into how the microecological distributions across various organs collectively influence gynecologic tumors. Most research has predominantly focused on the gut microbiome and digestive organs, leaving a gap in understanding the interactions between different microbial communities. However, studies on cervical cancer (CC) have highlighted the significant roles of human papillomavirus (HPV), vaginal microecology, and the intestinal microenvironment in disease progression, suggesting new therapeutic avenues (Teng et al., 2022). Understanding the interplay between these diverse microecologies can open new pathways for therapeutic interventions, particularly through targeted modulation of the microbiome to improve treatment outcomes for cervical cancer patients. The interactions among vaginal microbiota, gut microbiota, and systemic immune responses create a complex network that can either promote or inhibit tumor progression. This dynamic interplay is crucial for developing effective strategies against cervical cancer and could lead to innovative treatments that leverage the microbiome’s role in disease modulation.

Another research limitation is the gynaecologic cancer disparities reflect significant variations based on cancer type, ethnicities, and sociodemographic factors, necessitating a comprehensive examination of the underlying issues. This underscores the critical role of social determinants of health (SDOH), such as access to care, which significantly influence health outcomes (NIH HMP Working Group et al., 2009). For cervical cancer, timely screening and diagnostic evaluations are crucial; however, for ovarian and uterine cancers, emphasis must shift towards recognizing symptoms early and ensuring adherence to treatment guidelines, including biomarker testing. Addressing these disparities is essential for achieving health equity and improving outcomes for all patients affected by gynecologic cancers. This is not only patient care but also in research studies and clinical trials. There is a concerning underrepresentation of ethnic minority patients in randomized controlled trials (RCTs) for licensed systemic anti-cancer therapies (SACT) targeting gynecological cancers (Berg et al., 2020). In a recent review, analyzing 26 RCTs published between 2012 and 2022, found that 79.8% of the 17,041 participants were Caucasian, with significantly lower representation from East Asian (9.1%), Black/African American (3.7%), and other ethnic groups. The majority of research sites (80.1%) were located in North America, which limits enrollment opportunities for South Asian, Southeast Asian, and African populations. This disparity underscores the urgent need for the establishment of more research sites in underserved regions. Such actions are essential to ensure that findings are generalizable to diverse populations, ultimately contributing to health equity as cancer incidence continues to rise globally.

Another significant challenge lies in the technical methodologies employed across studies. Despite the promising advancements and successes in microbial genomics, the application of these techniques in clinical diagnostics has not kept pace, primarily due to a lack of regulatory frameworks and accreditation standards, as sequencing is not classified as a diagnostic procedure. Furthermore, clinical sequencing assays often lack universal reference standards and established methods for validating tests, ensuring reproducibility, and maintaining quality assurance. Most laboratories utilize Laboratory-Developed Tests (LDTs), which are specifically designed for clinical applications within a single clinical laboratory. These laboratories are certified under the Clinical Laboratory Improvement Amendments of 1988 (CLIA) and adhere to the regulatory standards established by CLIA for conducting high-complexity testing. Key obstacles to the widespread adoption of clinical microbial sequencing in advanced healthcare settings include issues related to cost, turnaround times, regulatory considerations, and, perhaps most importantly, the demonstrated clinical utility of these tests. Addressing these limitations is essential for integrating microbial sequencing into routine clinical practice and therapeutic.

## 7 Conclusion

In conclusion, the microbiome plays vital roles in preserving overall health. Over the past few years, studies have established connections between the microbial communities residing in the gastrointestinal tract and FRT and various diseases, including gynecologic cancers. Disruptions in the microbial composition of a microbiota, known as dysbiosis, promote cancer development. This is achieved through alterations in the immune response, disturbances in hormone metabolism, and modulation of the cell cycle, all of which contribute to a procarcinogenic environment. The application of omics technologies, such as metagenomics, metatranscriptomics, and metabolomics, presents significant opportunities for enhancing our understanding of functional microbiomes and metabolites and their interactions with host cells in the contexts of both health and disease. However, *in vitro* and *in vivo* investigations must support the hypotheses derived from omics studies. To study host–microbe interactions, experimental models that simulate the environments of the gastrointestinal tract and FRTs must be established. Such research efforts will yield valuable insights into potential therapeutic interventions. Moreover, the progress in developing and optimizing diagnostics, treatment strategies, drugs, probiotics, postbiotics, and vaginal flora transplantation is closely linked to these insights. To this end, it is essential to expand research efforts, employing molecular detection technologies in human samples, cells, and animal samples. These efforts will contribute to uncovering novel diagnostic and therapeutic targets for female reproductive diseases. The knowledge gained from these studies serves as a solid foundation for future research and holds great promise for the advancement of targeted therapeutic interventions, ultimately leading to improved clinical outcomes in gynecological cancers and related conditions.
